# Odimet^®^: A Pioneering Tele-Health Tool to Empower Dietary Treatment and the Acute Management of Inborn Errors of Metabolism—An Assessment of Its Effectiveness during the COVID Pandemic

**DOI:** 10.3390/nu16030423

**Published:** 2024-01-31

**Authors:** Paula Sánchez-Pintos, María José Camba-Garea, Beatriz Martin López-Pardo, María L. Couce

**Affiliations:** 1 Metabolic Diseases Unit, Neonatology Department, Clinical University Hospital of Santiago de Compostela, 15706 Santiago de Compostela, Spain; beatriz.martin.lopezpardo@sergas.es (B.M.L.-P.);; 2 IDIS—Health Research Institute of Santiago de Compostela, 15706 Santiago de Compostela, Spain; 3 Centro de Investigación Biomédica en Red Enfermedades Raras (CIBERER), Instituto Salud Carlos III, 28029 Madrid, Spain; 4 European Reference Network for Hereditary Metabolic Disorders (MetabERN), Via Pozzuolo, 330, 33100 Udine, Italy; 5 Faculty of Medicine, Santiago de Compostela University, 15704 Santiago de Compostela, Spain

**Keywords:** dietary program, inborn errors of metabolism, nutritional management, Odimet^®^, tele-health

## Abstract

Strict adherence to a diet is an essential pillar of long-term treatment for many inborn errors of metabolism (IEMs). Tools that educate patients about dietary management can positively condition adherence and prevent morbidity. We designed a free online dietary calculation program (Odimet^®^, version 2.1.) for IEMs patients in 2008, updated in 2022, that provides detailed information on the content of amino acids, protein, lipids, carbohydrates, vitamins and minerals in >3000 food products, including specific medical foods for IEM. We analyzed the statistics on visits to Odimet^®^ to evaluate its usefulness for long-term dietary management during a 5-year period focusing on three periods: pre-pandemic (15 March 2018–14 March 2020); pandemic 1 (15 March 2020–14 March 2021); and pandemic 2 period (15 March 2021–15 March 2023), in 120 patients with the following distribution: 84 patients with phenylketonuria (PKU); 12 with maple syrup urine disease (MSUD); 11 with urea cycle disorders (UCDs); and 13 with classical galactosemia. The evolutionary levels of their specific metabolic markers were evaluated, showing that globally, both pediatric and adult patients maintain a good metabolic control, even during a pandemic (median levels of phenylalanine in pediatric PKU patients 213.4 µmol/L and 482.3 µmol/L in adults; of leucine in MSUD patients: 144.2 µmol/L; of glutamine in UCDs: 726.8 µmol/L; and of galactose 1-phosphate levels in galactosemia: 0.08 µmol/L). The proportion of patients using Odimet^®^ ranges from 78–100%. An increase in the number of diets being calculated was observed during COVID-19 pandemic. Currently, 14,825 products have been introduced (3094 from the general database, and 11,731 added by users to their own profiles). In 2023 63 emergency dietary adjustments in the studied intoxication-type pathologies were calculated in Odimet^®^. Our results suggest that its regular use contributes to maintaining metabolic stability in IEMs patients, allowing them to adapt their menus to their lifestyle, and represents a powerful complementary tele-health tool which can be used to perform remote real-time dietary follow-up.

## 1. Introduction

The inborn errors of metabolism (IEMs) are a complex and heterogeneous group of more than 1450 genetic disorders [1], which are currently defined as being any condition in which the impairment of a biochemical pathway is intrinsic to the disease pathophysiology. IEMs include disorders due to primary enzyme defects and those associated with deficiencies of cofactors, transporters, chaperones, or transcription factors in metabolic pathways, as well as transporter superactivity. All ultimately result in a deficiency and/or accumulation of one or several metabolites [2]. Individual IEMs are rare or ultra-rare diseases, but collectively, they constitute frequent pathologies, with a global incidence of 1:800 to 1:2500 per live birth [3,4,5], and are an important cause of morbidity and mortality. The aim of dietary treatment for IEMs is to maintain metabolic stability and prevent the accumulation of toxic metabolites. Despite advances, dietary treatment, which mainly consists of life-long suppression, restriction or supplementation of one or several immediate principles combined with the administration of medical foods and/or supplements, is the mainstay of metabolic control [6,7,8,9,10,11,12]. This restrictive dietary therapy interferes with the desire for autonomy and the joy of eating and often results in a reduced quality of life and a lack of compliance [13]. The great heterogeneity of IEMs and their complex physiopathology necessitates individualized and dynamic dietary management that includes, during periods of metabolic stress (e.g., intercurrent illness), acute dietary readjustments to prevent metabolic decompensations, particularly in disorders with a risk of severe intoxication.

Strict adherence to dietary treatment is essential for maintaining optimal metabolic control and achieving adequate physical and neurological development. Encouraging adherence to a diet requires continual education, reinforcement and support from the family and professionals within the support team. The implementation of patient education is a recognized, feasible and cost-effective strategy that leads to better compliance to dietary and treatment recommendations for IEMs patients [14], as in other chronic diseases such as end-stage renal disease or diabetes [15,16,17]. Tools that facilitate nutritional calculation help patient instruction and contribute to reducing the lack of adherence to dietary treatment, a recognized issue in routine clinical practice, especially during adolescence and adulthood [18,19,20], avoiding nutritional imbalance and facilitating emergency dietary management.

Odimet^®^ is a free online dietary calculation program developed by our unit in 2008 and is specially adapted to the needs of IEM patients, although its use can be extended to the nutritional management of other types of pathologies.

The aims of the present study are: (I) to evaluate the effectiveness of this program in the clinical treatment of dietary basal and the acute management of IEMs, in order to maintain metabolic stability; and (II) to assess its utility as a telemedicine tool during the COVID pandemic for monitoring the diets of our patients, by comparing the number of diets calculated during the pandemic period, with greater difficulty of accessing in-person assistance, and the periods immediately before and after, during which the metabolic control visit regime was standard.

## 2. Materials and Methods

### 2.1. Odimet^®^ Characteristics

Odimet^®^ (https://www.odimet.es, accessed on 20 November 2023) is an open online dietary tool specifically designed for IEMs patients that provides detailed information on the content of amino acids (leucine, isoleucine, valine, phenylalanine, arginine, tyrosine, histidine, methionine, threonine, and tryptophan); proteins (total and natural); lipids (total fat content, saturated fats [C14:0, C16:0, C18:0], monounsaturated fats [C16:1, C18:1], polyunsaturated fats [C18:2, C18:3, C > 20], medium chain triglycerides [MCT], docosahexaenoic acid [DHA], eicosapentaenoic acid [EPA] and cholesterol); carbohydrates (total sugars, glucose, sucrose, fructose, lactose, galactose, maltose, starch and fiber); vitamins (vitamin A, B1, B2, B3, B6, B12, C, D, E, K); folic acid; and minerals (sodium, potassium, calcium, phosphorus, magnesium, iron, fluoride, copper, selenium, manganese, iodine and zinc) in 3094 food products, including natural foods, manufactured foods and, unlike other dietary calculation programs currently available, specific medicinal foods used for the management of IEMs.

The associated database is regularly updated and has the unique feature of allowing patients to add new products to their own profile. The following are the bibliographic sources used to gather nutritional information: USDA National Nutrient Database for Standard Reference (https://www.nal.usda.gov/, accessed on 15 July 2020); the National Food Institute of the Technical University of Denmark (https://www.food.dtu.dk/, accessed on 10 July 2022); BEDCA—the Food Composition Database of the Spanish agency for food safety and nutrition (https://www.bedca.net/, accessed on 15 July 2022); EuroFIR—the European Food Information Resource (https://www.eurofir.org/, accessed on 10 July 2022): the annual vade mecums of the manufacturers of specialized nutritional products for IEMs (Nutricia-SHS, Nestlé-Vitaflo, Mead Johnson, Lactalis-Sanutri, Orphan Europe, Cambrooke, Piam); and data sheets for low-protein products.

The program allows specific dietary calculation by meal (daily or weighted for several days); records previously calculated menus; details the contribution of total and natural protein, fats (total, saturated, and unsaturated fats) and carbohydrates, and the individual contribution of different amino acids and caloric intake; and can produce dietary reports detailing the patient’s weighted nutritional intake.

Access is free and password protected, guaranteeing privacy in accordance with Organic Law 15/1999 on the Protection of Personal Data. If users want the new products incorporated by themselves to be verified, it is necessary that they voluntarily share their own password with the metabolic team to access the program from their profile. Odimet^®^ is included in the register of intellectual property (registration number: 03/2008/924), and it was endorsed by the Interterritorial Council of the National Health System, which granted the program a Good Clinical Practices in Rare Diseases certification in 2013. (https://www.sanidad.gob.es/areas/calidadAsistencial/estrategias/enfermedadesRaras/BBPP/docs/-GALICIA_1.pdf, accessed on 12 January 2024). It was updated in November 2022 with the aim of: (i) improving accessibility by allowing its compatibility with mobile devices (smartphones and tablets) in order to facilitate access anywhere and at any time; (ii) enabling remote real-time review of menus to respond to the need for urgent interaction between patients and their care team to allow urgent dietary modifications; and (iii) facilitating its internationalization by incorporating an English version and the progressive incorporation of specific dietary products for EIM available in other countries.

### 2.2. Metabolic Control Assessment

To assess the utility of the program for dietary management in our IEMs patients, we evaluated the annual median levels, calculated over a 5-year period (March 2018–March 2023—subclassified in three periods: pre-pandemic (15 March 2018–14 March 2020); pandemic 1 (15 March 2020–14 March 2021); and pandemic 2 period (15 March 2021–15 March 2023), of the following metabolic parameters which were considered as markers of suitable metabolic control in the following conditions: plasma levels of phenylalanine (Phe) in phenylketonuria (PKU); leucine (Leu) in maple syrup urine disease (MSUD); ammonium and glutamine (Gln) in urea cycle disorders (UCDs); and galactose-1 phosphate (Gal 1-P) in dried-blood samples from classical galactosemia patients. We used the Phe target levels recommended in the European PKU Guidelines [7]: <360 µmol/L in children < 12 years of age and <600 µmol/L in children ≥ 12 years of age; a Leu target level of <380 μmol/L (<5.0 mg/dL); and the recommended Gln level of <1000 µmol/L in fasting state in accordance with UCDs guidelines [8]. A normal plasma concentration of Gal 1-P is <0.7 µmol/L, and normal ammonium levels are <110 µmol/L in newborns and <50 µmol/L in older children and adults [21].

Amino acid profile and hexoses monophosphate in blood samples were analyzed using MS/MS and an Applied Biosystems Sciex API 4000 apparatus [22,23]. Ammonium was determined using the enzymatic method AMM de Dimension^®^ (Siemens, Healthcare Diagnostics Inc., Newark, DE 19714, USA).

### 2.3. Website Traffic Analysis

We used Google analytics to analyze the traffic on the Odimet^®^ website. The following parameters were evaluated in the periods described: pageviews, defined as the number of pages that a user loads on the same website, which in Odimet, is equivalent to the number of diets calculated; number of sessions, defined as the period of time in which users interact with the website with inactivity of less than 30 min; the number of products included in the global database; and the number of products included by the users in the own profile.

A mapchart of Odimet® users was stablished by Google analytics (Universal Analytics, version 6 April 2023, accessed on 19 November 2023).

### 2.4. Statistical Analysis

Continuous variables were expressed as median and interquartile range for variables with skewed distribution. The non-parametric Mann–Whitney test was used after determining that data were significantly different from the normal distribution using the Kolmogorov–Smirnov test. Statistical significance was set at *p* < 0.05. Data were stored in Microsoft Excel (version 16.16.27.) data processor and statistical analyses and graphs were realized using Stata/IC version 16.1 software (StataCorp, College Station, TX, USA).

## 3. Results

### 3.1. Characteristics of the Study Population

A total of 120 patients were analyzed: 107 patients affected by aminoacidopathies (84 PKU patients; 12 MSUD patients; and 11 patients with UCDs), and 13 patients with classical galactosemia. As reflected in Table 1, which describes the characteristics of the study population, there is a predominance of adults among patients affected by PKU (66%), while in the rest of the studied pathologies the patients are mainly pediatric.

### 3.2. Metabolic Control

The proportion of patients controlled in our unit who use Odimet^®^ varies from 78% in PKU adult patients to 100% in MSUD, pediatric PKU patients and UCDs (Table 2). The regular employment of Odimet^®^ for dietary management contributes to an enhanced metabolic stability. As indicated in Table 2 and Table 3, both patients with aminoacidopathies, like PKU, MSUD or UCD, and patients with IEMs related to the metabolism of carbohydrates maintain a good level of metabolic control, with their metabolic markers remaining within the target levels during all of the study periods regardless of age, even during the pandemic. When comparing both pandemic periods with the pre-pandemic one, a statistical reduction was observed in Phe levels in pediatric and adult PKU patients (in adults in both pandemic periods in contrast to the pre-pandemic one and in children in pandemic period 2), in Gal 1-P levels in galactosemia patients and in glutamine levels in pandemic period 2.

The mean number of annual emergency dietary adjustments calculated by Odimet^®^ for the intoxication-type pathologies studied during the whole study period was 6.2 for MSUD patients and 2.8 for UCDs patients, with a total number of emergency diets calculated in 2023 (considering until 15 November 2023) of 63 (38 for MSUD patients and 25 for UCDs ones). An example of an ambulatory dietary adaptation in an MSUD patient during a febrile viral illness is detailed in Supplementary File S1.

Note that, in our unit, PKU and MSUD patients regularly send outpatient dried-blood samples for the determination of their Phe or branched chain amino acids levels, so the number of metabolic controls in those pathologies is clearly higher than in those pathologies that require control plasma samples such as UCD.

### 3.3. Website Traffic Analysis

The pageview registry, which represents the number of diets calculated using Odimet^®^, shows an increase in use throughout the three periods studied (Table 4) with a total of 257,799 diets calculated by the end of the pandemic 2 period. The number of users also increased to 3112 by 15 March 2023.

The number of sessions during the three periods was: 89,728 in the pre-pandemic period; 52,017 in pandemic period 1; and 116,054 in pandemic period 2; also showing an increase in use during the three periods (3023, 13,797 and 41,896 sessions/quarterly, respectively, in each period).

Currently, users have input data for 14,825 products; 3094 are products from the general database and 11,731 products have been added to their own profiles by users.

**Figure 1:**
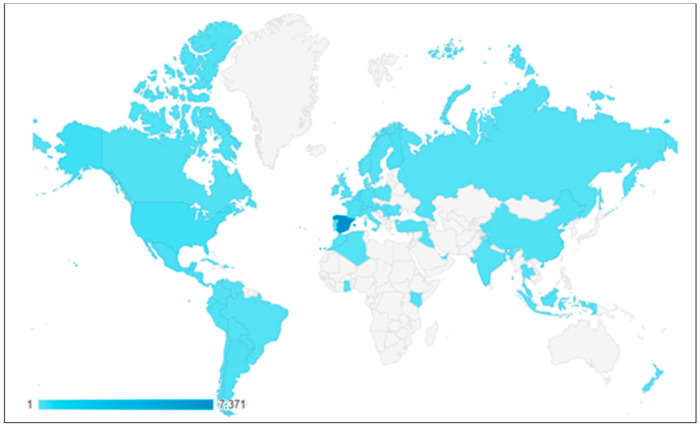
Geographic distribution of users since the launch of the new version. (Source: Google Analytics). Color intensity reflects the number of registered users in each country.

The user profile of the initial version was practically entirely Spanish, and to a lesser extent, Latin American; there has been a progressive increase in English users, and thanks to the current bilingual version, European and American users now represent 5.9% and 2.2%, respectively, of the total users. A graphical representation of the geographic distribution of Odimet^®^’s users is shown in Figure 1.

Analysis of data from 1 March 2018 to 15 March 2023 indicates that the preferred user device is a desktop computer (72.3%), followed by mobile phones (25.5%) and tablets (2.2%).

## 4. Discussion

Recent advances, including early diagnosis through neonatal screening programs, the development of specific therapies and improvements in dietary–nutritional treatment, mean that many IEMs are now considered “treatable rare diseases”. Dietary control is the main tool used for the therapeutic management of many IEMs, and continuous monitoring of nutritional and caloric intake is necessary to ensure adequate growth and development and avoid potential decompensation. Comprehensive nutritional management encompasses the development of support tools that facilitate nutritional calculation, continual education of patients about the dietary management of their disease and detailed dietary monitoring to achieve successful metabolic control and prevent decompensation.

There is a major unmet need for services that help patients with rare diseases to maintain their autonomy and self-manage their disease [24]. Factors known to affect dietary compliance in the transition into adulthood include leaving home, coping with the diet at work and maintaining a social life [25]. More specifically, a recent study found that the strongest self-reported negative impact on quality of life among young IEMs patients corresponded to relationships with friends and leisure activities, and was partly related to difficulties adapting a restricted diet during leisure activities or outings [26]. Tele-health-delivered dietary interventions offer feasible ways to facilitate dietary compliance, and when applicable, should be incorporated into health care services for the management of chronic conditions [27]. Odimet^®^ was specifically designed for the dietary management of IEMs and was developed to empower patients and their caregivers by enabling better dietary management through precise and dynamic dietary modifications which suit their specific condition and needs. It allows for the creation of varied and individualized diets that best suit the nutritional needs of each patient. The metabolic follow-up of our study population, showing a significant metabolic improvement in Phe, Gal 1-P and glutamine levels during the pandemic, reinforces the usefulness of Odimet in maintaining metabolic stability. In addition, Odimet^®^ facilitates real-time interactions between patients and their metabolic team to develop dynamic dietary modifications in cases of acute illness or surgery, as well as adaptions for specific conditions such as sport or stress. This feature markedly enhances patient/caregiver self-confidence in dietary management.

Intoxication-type IEMs have a major impact on patients’ health-related quality of life [28]. These patients deal with the permanent risk of metabolic crises that can be triggered by unpredictive circumstances, such as infections or stress, despite good metabolic control. This fear can negatively influence patient and caregivers´ psychological state or adaptive functioning [29,30]. Adjustments to acute situations require maintaining a dynamic cooperation between patients, families and the healthcare team [31]. However, this need is not adequately covered by healthcare services. In a recently published survey on the social and medical needs of patients with IEMs from the European Reference Network for Inherited Metabolic Disorders (Metab-ERN) [32], 30.5% of the patients consulted acknowledged difficulties in establishing contact with a member of their specialized metabolic team. This barrier was notably increased during the recent COVID pandemic, which strongly impacted the quality of life of chronic patients. To alleviate this difficulty in accessibility, the units were forced to carry out a rapid restructuring of clinical monitoring, making it clear that there is a need to have tools that allow for the remote monitoring of patients with the implementation of new telemedicine strategies. Data from the European Registry and Network for intoxication type metabolic diseases Consortium (E-IMD) indicate that regular follow-up visits for those patients were reduced by 41% during the pandemic, and the increased medical demand was mostly alleviated by remote technologies (86%) [33]. A key advantage of Odimet^®^ is that it facilitates real-time dietary supervision, enabling rapid and dynamic assistance in situations where there is a risk of metabolic decompensation, an aspect of particular interest in this type of IEMs. Our data indicate that, even though the percentage of our patients that employ Odimet^®^ is high (>78%), regardless of the pathology being studied, it reaches 100% among patients with intoxication-type IEMs (MSUD and UCDs patients). The number of emergency diets calculated confirms its usefulness for this purpose.

Like other remote medical assistance strategies which had a positive impact on IEMs patients during the pandemic [34,35], the program guaranteed continuous dietary supervision of our patients throughout lockdown periods, and allowed us to remotely implement emergency dietary adaptations in cases of acute illnesses. Odimet traffic analysis in the 5-year study period confirms the growing use of this tool. We consider the main indicator of website traffic the page visits, as this reflects the number of diets calculated. As reflected in Table 4, the evolutionary employment of Odimet^®^ shows an increase in the number of diets calculated during pandemic period 1, corresponding to the period of COVID confinement and the subsequent months, during which there was a progressive de-escalation of restrictions (p: not significant). This upward trend persists in pandemic period 2, once normal healthcare activity has been recovered. The number of sessions, an indicator of the amount of web traffic, also reflects an increase across the study period. Our experience indicates that Odimet^®^ is a powerful complementary tele-health strategy which facilitated remote clinical follow-ups which contributed to maintaining a continuity in care regardless of the epidemiological conditions. In particular, by allowing dynamic and real-time nutritional modifications, Odimet^®^ helped us address one of the most problematic issues for IEMs patients during lockdown period: the access to specialized healthcare.

Odimet^®^ is well known and widely used by health professionals in Spain and Spanish-speaking countries, and as of 2022, an English-language version has been available to facilitate its internationalization. Its versatility is demonstrated by its recommendation in the Nutritional Guide of the Spanish Pediatrics Association [36] and in the Spanish Ketogenic Diet Guide [37] and the extension of its use outside the scope of the IEMs, being a wide-ranging dietary–nutritional calculation tool which could be used for other chronic diseases [38,39].

Something which stands out as a strength of the study is the calculation of the percentage of patients who used the program, which was calculated directly by the researchers who knew which of the patients in our unit use this tool on a regular basis, given that Odimet^®^ allows for interactions between patients and/or their caregivers and the metabolic team, in order to avoid the risk of bias due to possible overestimation of the use of Odimet^®^ based on self-reported patient questionnaires. In the present study, only the most frequent IEMs which require dietary treatment at our unit were analyzed, which represents a methodological limitation.

## 5. Conclusions

Odimet^®^ is a simple, powerful, specific and dynamic tool for dietary calculation for IEMs that allows real-time remote dietary follow-up. Its increasing employment during the pandemic confirms its utility as a complementary tele-medicine tool used to maintain a continuity in care and metabolic stability, regardless of the epidemiological conditions.

## 6. Patents

Odimet has had an intellectual property registration since 26 June 2008 (registration number: 03/2008/924).

## Figures and Tables

**Table 1:** Characteristics of the study population.

IEMs (n)	Gender		Age	
	M	F	P	A
PKU (84)	36	48	28	56
MSUD (12)	6	6	12	0
UCDs (11)	5	6	7	4
Classic galactosemia (13)	5	8	13	0

P: ≤18 years; A: >18 years. A, adult; F, female; IEMs, inborn errors of metabolism; M, male; MSUD, maple syrup urine disease; n, number of patients; P, pediatric; PKU, phenylketonuria; UCDs, urea cycle disorders.

**Table 2:** Metabolic control and proportion of patients from each of the pathologies studied during the whole period who use Odimet^®^ (15 March 2018–15 March 2023).

IEM	Metabolic Marker	Mean [Interquartile Range]	% of Patients that Employ Odimet
PKU	Phe (µmol/L)		78–100%
	<12 y: vn < 360	251.46 [137.16–365.66]	
	≥12 y: vn < 600	365.76 [259.08–556.26]	
MSUD	Leu (µmol/L) (nv < 381)	175.2 [114.3–266.7]	100%
UCDs	Gln (µmol/L) (nv < 1000)	627 [518–820]	100%
	Ammonium (µmol/L) (nv < 50)	17 [10–25]	
Classic galactosemia	Gal 1-P (µmol/L) (nv < 0.7)	0.06 [0.03–0.1]	88%

Gal 1-P, galactose 1-phosphate; Gln, glutamine; IEM, inborn errors of metabolism; Leu, leucine; MSUD, maple syrup urine disease; nv, normal value; Phe, phenylalanine; PKU, phenylketonuria; UCDs, urea cycle disorders.

**Table 3:** Evolution of metabolic markers during the three study periods.

Metabolic Marker	Pre-Pandemic 15 March 2018–14 March 2020	Pandemic 1 15 March 2020–14 March 2021		Pandemic 2 15 March 2021–15 March 2023	
	Median [IQR] N	Median [IQR] N	*p* Value	Median [IQR] N	*p* Value
Phe (µmol/L)					
<12 y (nv < 360 µmol/L)	289.56 [190.50–381.00] N: 175	297.18 [228.6–411.48] N: 73	0.208	228.6 [121.92–358.14] N: 241	0.001
≥12 y (nv < 600 µmol/L)	411.48 [304.80–601.98] N: 203	365.76 [259.13–553.40] N: 130	0.022	358.14 [258.54–582.93] N: 316	0.021
Leu (µmol/L)					
(nv < 381 µmol/L)	167.64 [99.06–316.23] N: 515	175.26 [114.30–274.32] N: 290	0.540	175.26 [106.68–266.70] N: 647	0.872
Gln (µmol/L)					
(nv < 1000 µmol/L)	632.5 [538–890] N: 58	771.5 [644.5–1090] N: 28	0.002	567 [448–738] N: 63	0.018
Ammonium (µmol/L)					
(nv < 50 µmol/L *)	18 [12–27] N: 49	19 [10–27] N: 23	0.539	17 [10.5–22] N: 52	0.351
Gal 1-P (µmol/L)					
(nv < 0.7 µmol/L)	0.12 [0.08–0.27] N: 87	0.09 [0.05–0.17] N: 43	0.014	0.05 [0.03–0.09] N: 77	<0.001

The metabolic markers are expressed as median and interquartile range (between brackets). N reflects the number of determinations made of each marker. * All patients included were older than 1 year in the period studied (15 March 2018–15 March 2023). Gal 1-P, galactose 1-phosphate; Gln, glutamine; IQR, interquartile range; Leu, leucine; N, number of determinations; nv, normal value; y, years.

**Table 4:** Evolution of diets calculated using Odimet^®^ during the three study periods.

Period	N^er^ of Diets Calculated	Mean [Median] Pageviews/Quarterly	*p*	Pageviews/Session
*Pre-pandemic*	Total: 89,728	11,216 [11,292]		3.71
15 March 2018–14 June 2018	11,076			
15 June 2018–14 September 2018	9161			
15 September 2018–14 December 2018	13,531			
15 December 2018–14 March 2019	9999			
15 March 2019–14 June 2019	11,775			
15 June 2019–14 September 2019	8750			
15 September 2019–14 December 2019	13,927			
15 December 2019–14 March 2020	11,509			
*Pandemic 1*	Total: 52,017	13,004 [12,514]	0.2140	3.77
15 March 2020–14 June 2020	11,604			
15 June 2020–14 September 2020	10,262			
15 September 2020–14 December 2020	16,727			
15 December 2020–14 March 2021	13,424			
*Pandemic 2*	Total: 116,054	14,506 [14,661]	0.0913	2.77
15 March 2021–14 June 2021	13,094			
15 June 2021–14 September 2021	11,079			
15 September 2021–14 December 2021	13,267			
15 December 2021–14 March 2022	25,108			
15 March 2022–14 June 2022	16,041			
15 June 2022–14 September 2022	9460			
15 September 2022–14 December 2022	15,329			
15 December 2022–15 March 2023	12,676			

N^er^, number. *p*, comparation between the median of pandemic 1 and 2 with pre-pandemic period; significant if <0.05.

## Data Availability

The data that support the findings of this study are available from the corresponding author upon reasonable request.

## Supplementary Materials

The following supporting information can be downloaded at: https://www.mdpi.com/article/10.3390/nu16030423/s1, File S1 shows an example of emergency dietary adaptation in a patient with Maple syrup urine disease during a febrile illness.
