# Supplementary material for: Odimet®: A Pioneering Tele-Health Tool to Empower Dietary Treatment and the Acute Management of Inborn Errors of Metabolism—An Assessment of Its Effectiveness during the COVID Pandemic
Source: Nutrients. 2024 Jan 31;16(3):423. doi: 10.3390/nu16030423 (PMC10856987; doi:10.3390/nu16030423)
Supplement: Supplementary file 1 [file nutrients-16-00423-s001.zip › nutrients-2776584-supplementary.pdf]

Normal Diet Id.74500

NAME: MSUD PATIENT

AGE: 0 WEIGHT: 21 kg HEIGHT: 0 cm IMC: 0 DATE: 2022-04-26

CONTENT

DIET PRODUCTS		
CHICKPEA (15 g)	MACARONI (SANAVI) (95 g)	OLIVE OIL (10 g)
OLIVE (40 g)	PUMPKIN (90 g)	CANNED ASPARAGUS (20 g)
COOKED POTATO (70 g)	SWEET CORN, YELLOW, CANNED (35 g)	SOLIS FRIED TOMATO (30 g)
EROSKI MUSHROOMS (45 g)	MSUD ANAMIX JUNIOR (108 g)	VEGAN CHEESE VIOLIFE MOZZARELLA FLAVOR SLICES (30 g)

DIET CALCS

VARIABLE	kCal	%
**Proteins**	154.4	13.37
**Fats**	333.36	28.86
**Carbohydrates**	667.28	57.77
**Energetic value**	1155.04	

VARIABLE	VALUE
**Kcal/ml (Liquid Vol.: 0 ml)**	0
**Kcal/Kg (Weight: 21 Kg)**	55
**Taotal proteins/Kg**	1.84
**Natural proteins/Kg**	0.4

FATS DISTRIBUTION	%
**Saturated**	8.38
**Monounsaturated**	15.26
**Polyunsaturated**	3.05

COMPONENTS

PROTEINS	
**Total proteins**	**38.6 g**
**Natural proteins**	**8.36 g**
Total Nitrogen	6.19 g
Valine	290.85 mg
Leucine	431.7 mg
Isoleucine	256.6 mg
Phenylalanine	2328.4 mg
Tryptophan	671.35 mg
Lysine	2731.15 mg
Arginine	2200.8 mg
Histidine	1127 mg
Methionine	910.55 mg
Threonine	3089 mg
Tyrosine	2917.95 mg

FATS	
**Total fat**	**37.04 g**
**Saturated**	**10.75 g**
C 14:0	0.01 g
C 16:0	1.77 g
C 18:0	0.3 g
**Monounsaturated**	**19.58 g**
C 16:1	0.17 g
C 18:1	10.5 g
**Polyunsaturated**	**3.92 g**
C 18:2	1.86 g
C 18:3	0.14 g
**C>20**	**0 g**
**MCT**	**0.58 g**
**DHA**	**0 g**
**EPA**	**0 g**
**Cholesterol**	**0.67 mg**

CARBOHYDRATES	
**Carbohydrates**	**166.82 g**
Total sugars	10.31 g
Sucrose	1.52 g
Glucose	0.36 g
Fructose	0.33 g
Lactose	0 g
Galactose	0.03 g
Maltose	0.1 g
Starch	0 g
Fiber	19.01 g

MINERALS	
Sodium	886.04 mg
Potassium	1291.05 mg
Calcium	1244.29 mg
Phosphorus	914.79 mg
Magnesium	184.09 mg
Iron	15.48 mg
Fluoride	0 mcg
Copper	1.17 mg
Selenium	35.92 mcg
Manganese	1.67 mg
Iodine	150.49 mcg
Zinc	7.01 mg

VITAMINS	
Vitamin A	803.4 mcg
Vitamin B1	1 mg
Vitamin B2	0.97 mg
Vitamin B3	4.38 mg
Vitamin B6	1.14 mg
Folates	363.55 mcg
Vitamin B12	2.15 mcg
Vitamin C	65.21 mg
Vitamin D	22.68 mcg
Vitamin E	12.32 mg
Vitamin K	33.77 mcg

Normal Diet Id.78457

NAME: MSUD. Emergency diet treatment during viral infection

AGE: 7 WEIGHT: 21 kg HEIGHT: 118 cm IMC: 15.08 DATE: 2022-06-28

CONTENT

DIET PRODUCTS		
MACARONI (SANAVI) (95 g)	SOLIS FRIED TOMATO (30 g)	VEGAN CHEESE VIOLIFE MOZZARELLA FLAVOR SLICES (30 g)
OLIVE OIL (40 g)	EROSKI MUSHROOMS (45 g)	PROZERO (DAIRY SUBSTITUTE) (500 g)
PUMPKIN (90 g)	MSUD ANAMIX JUNIOR (125 g)	

DIET CALCS

VARIABLE	kCal	%
**Proteins**	151.24	9.34
**Fats**	743.49	45.9
**Carbohydrates**	725.08	44.76
**Energetic value**	1619.81	

VARIABLE	VALUE
**Kcal/ml (Liquid Vol.: 0 ml)**	0
**Kcal/Kg (Weight: 21 Kg)**	77.13
**Taotal proteins/Kg**	1.8
**Natural proteins/Kg**	0.13

FATS DISTRIBUTION	%
**Saturated**	13.33
**Monounsaturated**	25.53
**Polyunsaturated**	4.14

COMPONENTS

PROTEINS	
**Total proteins**	**37.81 g**
**Natural proteins**	**2.81 g**
Total Nitrogen	6.1 g
Valine	31.5 mg
Leucine	41.4 mg
Isoleucine	27.9 mg
Phenylalanine	2386.4 mg
Tryptophan	710.8 mg
Lysine	2773.6 mg
Arginine	2073.6 mg
Histidine	1139.4 mg
Methionine	959.9 mg
Threonine	3351.1 mg
Tyrosine	3187.8 mg

FATS	
**Total fat**	**82.61 g**
**Saturated**	**23.99 g**
C 14:0	0.01 g
C 16:0	4.55 g
C 18:0	0.78 g
**Monounsaturated**	**45.94 g**
C 16:1	0.51 g
C 18:1	28.52 g
**Polyunsaturated**	**7.46 g**
C 18:2	3.9 g
C 18:3	0.3 g
**C>20**	**0 g**
**MCT**	**0.68 g**
**DHA**	**0 g**
**EPA**	**0 g**
**Cholesterol**	**0.67 mg**

CARBOHYDRATES	
**Carbohydrates**	**181.27 g**
Total sugars	24.94 g
Sucrose	0 g
Glucose	0 g
Fructose	0 g
Lactose	0 g
Galactose	
Maltose	0 g
Starch	0 g
Fiber	14.84 g

MINERALS	
Sodium	725.9 mg
Potassium	928.5 mg
Calcium	1373.9 mg
Phosphorus	924.6 mg
Magnesium	164.55 mg
Iron	14.35 mg
Fluoride	0 mcg
Copper	0.89 mg
Selenium	38.77 mcg
Manganese	1.36 mg
Iodine	173.89 mcg
Zinc	6.92 mg

VITAMINS	
Vitamin A	857.1 mcg
Vitamin B1	0.96 mg
Vitamin B2	1.01 mg
Vitamin B3	3.04 mg
Vitamin B6	0.96 mg
Folates	276.9 mcg
Vitamin B12	2.38 mcg
Vitamin C	60.6 mg
Vitamin D	26.25 mcg
Vitamin E	17.19 mg
Vitamin K	42.57 mcg
